# Multimodal approach for surgical site infection prevention – results from a pilot site in Kenya

**DOI:** 10.1186/2047-2994-4-S1-P87

**Published:** 2015-06-16

**Authors:** P Ntumba, C Mwangi, J Barasa, A Aiken, Z Kubilay, B Allegranzi

**Affiliations:** 1 AIC Kijabe Hospital, Kijabe, Kenya; 2 Service Delivery and Safety, World Health Organization (WHO), Geneva, Switzerland

## Introduction

Surgical Site Infections (SSI) are the most frequent healthcare-associated infection in developing countries with incidence rates up to 30%. Prevention of SSI is complex and faces multiple challenges, especially in resource limited settings. Since 2013, WHO in collaboration with Johns Hopkins University, has been leading the Surgical Unit Safety Program (SUSP) in 5 African hospitals, one of which is the AIC Kijabe Hospital, a private teaching facility in rural Kenya.

## Methods

The SUSP intervention incorporated a bundle of 6 SSI prevention measures selected as priority by the site leaders (pre-operative bathing, avoiding hair removal, optimal surgical hand and skin preparation using locally produced alcohol-based products, appropriate surgical antibiotic prophylaxis and improving operating room discipline) embedded within adaptive work to improve the safety culture. Implementation was achieved with local adaptation and creation of tools for advocacy, training, leadership and front-line staff and patient engagement. SSI surveillance and process measures evaluation reflecting the intervention have been carried out throughout the study period for about 18 months, based upon a WHO protocol using standardized definitions.

## Results

Preliminary data show that the crude SSI rate significantly decreased from 9.3% (38/406 patients) before to 5% (18/353) post-intervention. Patients receiving post-operative antibiotics decreased from 50% to 26%; hair removal with shaving decreased from 25% to 2% of patients; theatre discipline improved with a drop in the average number of door openings per operation from 55 to 40.

## Conclusion

Implementation of a SSI prevention bundle and creation of a safety climate was successfully achieved at AIC Kijabe Hospital with tangible reductions in SSI rates and improvement of process measures. Local production of alcohol-based products for surgical hand and surgical site preparation was an innovative approach to overcome availability and cost barriers. Engagement of senior staff coupled with structured management of patient safety programs helped inculcate these concepts into the local culture and practice and are crucial for the long term sustainability.

## Disclosure of interest

None declared.

